# Retraction: Kinesin family member 2A serves as a potential biomarker reflecting more frequent lymph node metastasis and tumor recurrence risk in basal-like breast cancer patients

**DOI:** 10.3389/fsurg.2023.1307334

**Published:** 2023-10-11

**Authors:** Frontiers Editorial Office

A Retraction of the Original Research Article Kinesin family member 2A serves as a potential biomarker reflecting more frequent lymph node metastasis and tumor recurrence risk in basal-like breast cancer patients By Yang H and Liu Y. (2022) Front. Surg. 9:889294. doi: 10.3389/fsurg.2022.889294

The journal and Chief Editors retract the 16 June 2022 article cited above.

This article is retracted by Frontiers. The publisher has discovered that the authors provided false information for the peer-review process. As the scientific integrity of the article cannot be guaranteed, and adhering to the recommendations of the Committee on Publication Ethics (COPE), the publisher therefore retracts the article.

The authors have not responded to this retraction.

This retraction was approved by the Chief Editors of Frontiers in Surgery and the Chief Executive Editor of Frontiers.

